# Editorial: Innovative and creative behaviours in the modern workplace: causes and consequences

**DOI:** 10.3389/fpsyg.2025.1647616

**Published:** 2025-07-04

**Authors:** Sheng Huang, Mike James Mustafa, Mathew Hughes, Delia Virga

**Affiliations:** ^1^Institute of Work, Organisation and Wellbeing, University of Nottingham Malaysia, Semenyih, Malaysia; ^2^School of Business, University of Leicester, Leicester, United Kingdom; ^3^Department of Psychology, Universitatea de Vest din Timişoara, Timişoara, Romania

**Keywords:** creativity, innovative behavior, innovation, culture, creative behavior

Rapid technological development has created the need for bold, radical changes (Dwivedi et al., 2023), rendering the future more unpredictable and uncertain. Consequently organizations and individuals must continuously adapt to such situations to remain competitive (Gu et al., 2023). Specifically, individual creative and innovative actions are becoming increasingly important for organizational competitiveness and success (Rietzschel et al., 2024; Woods et al., 2018). This Research Topic explores novel and useful theoretical approaches to enriching our understanding of the mechanisms and processes through which creative and innovative behaviors emerge in organizations.

Receiving over 67 submissions in total, the 10 carefully selected works (see Table 1) represent various countries in Asia and diverse samples, such as general employees, elder workers, organizational leaders, and entrepreneurs. The studies span multiple sectors, including manufacturing, public services, and knowledge-intensive industries, thus providing nuanced insights into the multifaceted drivers of employee creative and innovative behaviors in modern workplaces. By highlighting commonalities and contextual differences, the studies offer valuable guidance for organizations and leaders seeking to foster creativity and innovation from within.

**Table 1:** Overview of selected articles.

**Creative and innovative behaviors in the modern workplace: causes and consequences**									
**No**	**Authors**	**Research questions**	**Num**	**Participants and country**	**Key variables studied**	**Findings**	**Theories applied**	**Theoretical contribution**	**Practical contribution**
1	Wang L. et al.	How does leader perfectionism affect radical innovation?	343	Employees of SEMs, China	Leader perfectionism, Leader's conscientiousness, work engagement, promotion focus, radical innovation	Work engagement mediates; conscientiousness and promotion focus moderate.	JD-R theory,	Found that leader's other-oriented perfectionism boosts radical innovation via work engagement.	Incorporated both leader and follower traits in understanding innovation dynamics.
2	Li Y. et al.	How does craftsmanship spirit influence innovative behavior?	400	Skilled workers, Manufacturing, China	Craftsmanship spirit, Innovative self-efficacy, knowledge sharing, Innovative climate, Innovative behavior,	Mediated by self-efficacy and knowledge sharing; innovative climate as moderator.	Social Cognitive Theory: Social Exchange Theory:	Bridges mindset and behavior through dual mediators. Promoted the concept of craftsmanship as a psychological driver of innovation.	Emphasizes crafting spirit in HR practices.
3	Jiang B. et al.	How does overqualification relate to innovation performance via perfectionism and job crafting?	363	Employees, manufacturing and service, China	Perceived overqualification, Independent self-construction, perfectionism, job crafting, informal status, innovation performance	Chain mediation via perfectionism and job crafting; moderated by independent self-construction and informal status.	Trait Activation Theory	Shows double mediation and dual moderation effect. Framed perceived overqualification as a source of innovation via perfectionism and job crafting.	Reframes overqualification as a creative resource.
4	Lee and Kim	What drives innovative behavior in public organizations?	1,021	Public servants, South Korea	Public service motivation, organizational commitment, perceived innovative culture Innovative behavior	PSM drives innovation; organizational commitment not mediating; perceived innovative culture moderates PSM and innovation behavior.	Public Service Motivation Theory	Clarifies PSM and innovative culture effects on innovative behavior in public sector. Confirmed that PSM directly fosters innovation even when organizational commitment does not mediate.	Recommends PSM-based recruitment and cultural alignment. Showed that innovative culture strengthens the impact of motivation on innovation.
5	Yang et al.	How does entrepreneur creativity transform into organizational creativity?	458	Entrepreneurs and organization members, China	Entrepreneur creativity, platform leadership, org culture(moderator) Org creativity	Platform leadership mediates, org culture moderates transformation.	Social Exchange Theory, Social information processing theory Social cognitive theory	Extends understanding of top-down creativity transfer. Highlighted that platform leadership enables the transfer of entrepreneurial creativity to organizations.	Guides leaders to facilitate creativity diffusion.
6	Jiang D. et al.	How do HIWPs influence elder employees' innovation performance?	278	Elder employees (>35), China	HIWPs, exploratory and exploitative innovation, transformational leadership innovation performance	Non-linear U-shaped and inverted U-shaped effects observed; transformational leadership moderates.	Social Exchange Theory Self-determination theory	Discovered nonlinear U-shaped and inverted-U effects of innovation strategies on older employees. Reveals complex dynamics of HIWPs and innovation forms.	Offers HR strategies for elder employees' innovation.
7	Liu et al.	How does humble leadership affect creative performance?	350	Employees and supervisors, Pakistan	Humble leadership, intrinsic motivation, work engagement creative performance	Intrinsic motivation and work engagement sequentially mediate humble leadership–creativity link.	Self-Determination Theory, JD-R model	Identified sequential mediation of intrinsic motivation and work engagement in creativity. Advanced leadership research by showing humble leadership's motivational power.	Promotes humble leadership as a creativity enabler.
8	Arshad et al.	How does HPWS impact innovation via social capital and knowledge-sharing?	262	Job incumbents, service firms, Pakistan	HPWS, social capital, knowledge sharing, need for cognition, Innovative behavior	HPWS promotes innovation via social capital and knowledge sharing; need for cognition moderates.	Social Exchange Theory, Elaboration Likelihood Model	Combines social/cognitive pathways to explain innovation behavior. Showed that social capital and knowledge sharing mediate the HPWS-innovation link.	Guides design of HPWS to foster innovation in knowledge firms.
9	Wang C. et al.	Does organizational ambidexterity mediate high performance HR practices - performance link? Does organizational learning moderate the organizational ambidexterity- performance link?	347	Senior managers of SMEs, China	HPHR practices, organizational ambidexterity, organizational learning Organizational performance	HPHR improves organizational performance; ambidexterity mediates; learning moderates.	Strategic human resource management theory, strategic management theory, contingency theory, organizational learning theory	Clarifies how organizational ambidexterity mediates HPHR practices- organizational performance relation. Revealed that organizational learning enhances the effect of HR practices through ambidexterity.	Suggests organizations to realize organizational ambidexterity by integrating HPHR practices, ultimately enhance organizational performance.
10	Li S. et al.	How can nudging push employees beyond comfort zones in green innovation?	2,253	Employees from manufacturing firms, China	Combination nudge, isolation nudge, green knowledge acquisition	Combined nudges (social norm + status) outperform individual ones; praise-before-pressure more effective.	Expectancy Theory	Expands expectancy theory in green learning via nudging mechanisms. Demonstrated that combining praise and social norm nudges is more effective than using pressure.	Offers effective nudging strategies for green innovation.

Broadly, the 10 articles illustrate the diverse psychological, organizational, and contextual mechanisms that underpin employee creative and innovative behaviors in the modern workplace. The studies by Jiang D. et al., Arshad et al., and Wang C. et al. demonstrate how human resource (HR) practices can play a pivotal role in shaping organizational innovation. These practices include high-involvement work practices, high-performance work systems, and high-performance HR practices. Additionally, other mechanisms, such as fostering intrinsic motivation (Liu et al.), promoting knowledge sharing (Li Y. et al.), and facilitating knowledge acquisition (Li S. et al.), are identified as effective strategies that managers and HR professionals can employ to cultivate creativity and innovation, even in the absence of technical backgrounds. Moreover, research by Li Y. et al., Lee and Kim, and Yang et al. suggests that the organization's innovative climate and culture have conditioning effects on both actor-level and contextual factors that shape employees creative and innovative behaviors. Specifically, supportive climates and cultures positively condition these relationships, strengthening their impact on creative and innovative behavior. Collectively, such findings offer valuable insights and practical approaches that can guide HR professionals in fostering organizational creativity and innovation from a non-technical perspective.

Notably, the studies also reveal moderated mediation mechanisms through which HR practices and leadership styles influence creative and innovative behavior, including organizational learning (Wang C. et al.), transformational leadership and leader's conscientiousness (Jiang D. et al.; Wang L. et al.). Jiang D. et al. examined nonlinear and cross-level dynamics, uncovering the nuanced relationships between factors such as organizational ambidexterity, transformational leadership, and innovation outcomes.

From a theoretical prestige, four articles utilized relational and exchange-based theories (Li Y. et al.; Yang et al.; Jiang D. et al.; Arshad et al.). This may hold particular significance for employers and managers who need to recognize this common interest in the workplace, as it suggests that individuals value reciprocation when they make efforts to contribute to the organization. One cannot expect employees to deliver top performance without providing them with adequate resources and appropriate rewards—such an expectation is akin to wanting to have one's cake and eat it too. The remaining articles drew on a broad range of psycho-social theories such as job demands—resources (JD-R) theory (Wang L. et al.; Liu et al.), trait activation theory (Jiang B. et al.), expectancy theory (Li S. et al.), self-determination theory (Liu et al.; Jiang D. et al.), and social cognitive theory (Li Y. et al.; Yang et al.) to explore how employees' creative and innovative behaviors are supported in the workplace.

The Research Topic contributes theoretically in two main ways: it combines various levels of analysis—individual, team, and organizational—to offer a comprehensive understanding of the factors leading to creativity and innovation. Additionally, they connect psychological and strategic viewpoints by correlating intrinsic motivation and cognition with performance outcomes at the firm level. From a practical standpoint, the findings suggest actionable strategies for organizations, managers, HR professionals such as creating tailored HR systems that signify commitment to employees; cultivate leadership that promotes motivation and engagement; acknowledge and utilize individual differences like over qualification or a craftsman spirit; and nurture organizational cultures that fostering knowledge sharing and learning, building social capital, and deploying behavioral nudges to enhance creativity and innovation. Such studies bridge theoretical gaps and provide a solid foundation for practical implementation in creativity- and innovation-driven organizations.

While the Research Topic provides solid and comprehensive contributions to both theory and practice, further research is still needed. For instance, studies have shown individuals' psychological capital significantly enhances employee performance, including employee creativity and innovation (Ghafoor and Haar, 2022; Tho, 2022). Creativity and innovation are highly cognitively demanding processes that consume personal resources (Serban et al., 2023; Sidelkivska and Bilbao-Calabuig, 2023), while psychological capital is a major individual psychological resource that acts as a major personal resource in support of employee creative and innovative behavior (Ghafoor and Haar, 2022). It encompasses hope, optimism, self-efficacy, and resilience, which are considered fundamental drivers of creative and innovative behavior (Luthans and Youssef-Morgan, 2017; Newman et al., 2014; Yu et al., 2019). However, there is a lack of understanding regarding the contexts and conditions that underlie the relationships between psychological capital, creativity, and innovation (Loghman et al., 2023; Lupsa et al., 2020). Accordingly, future research would benefit from examining how and when psychological resources can promote creative and innovative behavior in the workplace. In particular, further research is needed to gain a deeper understanding of how proximal factors in an employee's work environment, such as their job, either challenge or hinder their ability to engage in creative and innovative behaviors (De Clercq and Mustafa, 2024). Such efforts can advance our understanding of the conditions and boundaries that facilitate or inhibit the effective translation of psychological resources into creativity and innovation.

In the era characterized by rapid technological change, heightened unpredictability, and growing uncertainty, literature increasingly highlights creativity and innovation as vital drivers of organizational adaptability and long-term success (Sidelkivska and Bilbao-Calabuig, 2023; Zhou and Hoever, 2023). Advancing our understanding of creative and innovative behavior is essential for supporting employers, managers, and HR professionals in navigating these complex challenges. However, the number of empirical research studies in this Research Topic remains limited. Therefore, further scholarly efforts are warranted to conduct empirical studies to support and enable organizations to remain competitive and future-ready.
